# Effects of a School-Based Intervention on Executive Functions and Theory of Mind in Children with Specific Learning Disorders

**DOI:** 10.3390/brainsci16010042

**Published:** 2025-12-28

**Authors:** Stella Tsermentseli, Aikaterini Pavlidou, Evangelia-Chrysanthi Kouklari

**Affiliations:** 1 Developmental & Educational Psychology Laboratory, Pedagogical Department of Primary Education, University of Thessaly, 382 21 Volos, Greece; pavlidou.kat@gmail.com; 2 Department of Child Psychiatry, Aghia Sophia Children’s Hospital, School of Medicine, National and Kapodistrian University of Athens, 115 27 Athens, Greece; evakouklar@gmail.com

**Keywords:** executive functions (EF), Theory of Mind (ToM), Specific Learning Disorders (SLD), school-based intervention, cognitive training

## Abstract

**Background/Objectives:** Executive functions (EFs) and Theory of Mind (ToM) are often compromised in children with Specific Learning Disorders (SLD). Although evidence highlights the malleability of EF, studies have yet to investigate whether school-based interventions can enhance both cool and hot EF domains and support ToM development in this population. This study evaluated the effectiveness of a structured, classroom-based EF training program in improving cool EF, hot EF, and ToM in children with SLD. **Methods:** Forty students with SLD (aged 8–10 years) were allocated to an intervention group (*n* = 24) or a passive control group (*n* = 16). The program was delivered in small groups during regular school hours over 6–9 weeks (18 sessions). Pre- and post-intervention assessments measured cool EFs (working memory, planning, cognitive flexibility, inhibition), hot EFs (affective decision-making, delay of gratification), and ToM (false belief understanding, mental state/emotion recognition). **Results:** The intervention group showed significant within-group improvements in working memory, planning, and cognitive flexibility, whereas the control group showed no significant changes. Between-group comparisons revealed significant effects for working memory, planning, and ToM mental state/emotion recognition, with medium-to-large effect sizes. No significant group differences were found for hot EFs or ToM false belief understanding. **Conclusions:** These findings suggest that participation in a structured, school-based EF program is associated with selective improvements in specific cool EF components and one aspect of ToM in children with SLD, supporting the potential value of classroom-based interventions for cognitive and socio-cognitive development.

## 1. Introduction

Specific Learning Disorders (SLD) are neurodevelopmental conditions characterized by persistent difficulties in learning and using academic skills, such as reading, writing, or mathematics, that are substantially below what is expected for the individual’s age and interfere with academic or occupational functioning [1]. Beyond their academic impairments, SLDs are increasingly understood as involving underlying neuropsychological dysfunctions, particularly within the domain of executive functions (EFs) [2,3]. EFs refer to top–down, self-regulatory cognitive processes that support goal-directed behavior and adaptive learning [4,5]. Contemporary models distinguish between “cool” EFs (e.g., working memory, inhibition, cognitive flexibility, planning) which operate in abstract, affect-neutral contexts, and “hot” EFs, which involve affective or motivational salience (e.g., affective decision-making, delay of gratification) [6]. These capacities rely on partly overlapping neural systems, with cool EFs associated primarily with dorsolateral prefrontal and frontoparietal networks, whereas hot EFs depend more on ventromedial and orbitofrontal cortices and their connections to limbic structures such as the amygdala and striatum [6,7].

A substantial body of research indicates that children with SLD present with difficulties across cool EF domains, including working memory, inhibition, and cognitive flexibility [8,9,10]. Although such deficits are consistently reported, findings remain heterogeneous, suggesting domain-specific profiles rather than a uniform deficit. More recently, research has begun to explore whether the EF difficulties associated with SLD extend beyond cool EF domains to encompass affective hot EF and social-cognitive functioning. Emerging evidence suggests that children with SLD may experience weaknesses in hot EF—specifically delay of gratification—as well as in Theory of Mind (ToM), the ability to infer others’ mental and emotional states [11].

Although a growing body of research has documented associations between executive functions and ToM, this relationship is neither uniform nor linear across development or clinical populations. While cool EF components such as working memory, inhibition, and cognitive flexibility have been shown to correlate with ToM performance in both typically developing children and neurodevelopmental conditions, findings remain heterogeneous, and the directionality and mechanisms underlying these associations are still debated [12,13,14,15]. In particular, evidence in children with SLD is limited and mixed, with some studies reporting EF–ToM associations and others suggesting dissociable developmental trajectories [11]. These inconsistencies indicate that EF may function as a facilitating or constraining condition for ToM reasoning rather than as a direct causal determinant.

From a developmental perspective, EF and ToM are increasingly conceptualized as partially overlapping, dynamically interacting systems that may influence one another indirectly through shared cognitive, neural, and environmental factors, including language, metacognition, and social interaction demands [12,13]. Neuroimaging findings further suggest overlapping, but not identical, neural substrates, particularly within prefrontal and temporoparietal regions, supporting the view of a complex, non-linear relationship rather than a simple direct effect [14]. Accordingly, improvements in EF may create more favorable conditions for ToM engagement without necessarily resulting in uniform or immediate gains across all ToM components.

Within the context of heterogeneity and theoretical debate, cool EF components, particularly working memory and inhibition, have been found to predict ToM performance, supporting theoretical accounts positing that EF capacities may serve as a scaffold, rather than a direct causal driver, for ToM reasoning [12,13]. Neuroimaging studies further document overlapping activation patterns across EF and ToM networks, especially within prefrontal and temporoparietal regions [14,15]. Taken together, these findings suggest that EF deficits in SLD may constrain not only academic functioning but also socio-cognitive development, highlighting the importance of addressing EF difficulties in SLD, as downstream effects may manifest in socio-cognitive domains relevant for classroom adaptation and peer interaction.

In response to these challenges, EF-based interventions have gained prominence as a promising avenue for supporting children with SLD [16,17]. Over the past two decades, diverse EF intervention models have been developed, including computerized working-memory programs, cognitive-rehabilitation protocols, and classroom-based curricula [18,19,20,21,22,23,24,25,26,27,28,29,30,31]. Existing evidence indicates that EF interventions can lead to improvements in targeted EF components and far-transfer to reading, spelling, and numeracy performance. Working memory training, for instance, has been associated with gains in reading and math fluency [20,21,22], while interventions targeting inhibition and cognitive flexibility have shown enhancements in attentional control and academic perseverance [23]. Meta-analytic findings indicate that multicomponent EF interventions implemented in ecologically valid educational contexts yield the most robust and transferable effects [23,24].

Building on this evidence base, intervention studies in SLD populations have adopted various formats. Computerized approaches, including virtual reality platforms, tele-rehabilitation systems, and game-based training, have demonstrated moderate-to-large improvements in working memory, inhibition, and cognitive flexibility, with some evidence for transfer to literacy and numeracy and modest long-term maintenance [23,26,27]. Long-term generalization remains inconsistent; some programs yield selective improvements in specific EF components [19] (e.g., visuospatial memory), while others produce broader EF gains [28]. Cognitive rehabilitation studies have likewise reported robust improvements in EF and reading-related skills, demonstrating the neuroplastic potential of EF training [22,29,30]. Increasingly, evidence highlights the effectiveness of ecologically valid, classroom-embedded EF programs that support active engagement, repeated practice, and integration with school activities [30,31].

Yet substantial challenges remain. Many existing EF programs are highly individualized, rely heavily on digital delivery, or lack ecological validity and opportunities for generalization. To our knowledge, no study to date has examined whether EF training can produce far transfer to ToM or broader social-cognitive abilities in children with SLD, despite strong theoretical and neurobiological links between these domains. Interventions explicitly targeting both cognitive and affective of EF remain rare, underscoring the need for school-based programs that integrate EF development within naturalistic, collaborative learning environments.

The present study evaluated a structured, classroom-based EF training program designed to strengthen cognitive flexibility, goal setting, and problem-solving through collaborative, developmentally appropriate activities. The overarching aim was to examine whether participation in the intervention enhanced both cool and hot EF skills in primary school children with SLD and whether these gains extended to ToM. Based on prior evidence indicating consistent deficits in cool EF domains and their responsiveness to training [24,31], it was hypothesized that children in the intervention group would show greater improvements in cool EF compared with controls. Although the program utilized mainly trains cool EF skills, its delivery in socially interactive, emotionally meaningful classroom contexts could engage hot EF, thereby allowing for the possibility of small to modest gains in these processes. A further exploratory aim was to determine whether EF improvements would generalize to ToM abilities, including mental state/emotion recognition and false-belief reasoning, though no directional hypothesis was advanced for this. Although previous studies suggest correlational links between EF and ToM in typical and atypical populations [11,32], the available evidence remains insufficient to support a clear mechanistic account of EF–ToM transfer in SLD populations. As the intervention did not directly train social-cognitive strategies, no directional hypothesis was formulated for ToM. Instead, ToM was included as an exploratory outcome to examine whether indirect transfer effects might emerge. By embedding EF training within naturalistic classroom settings and assessing both near-transfer (EF) and far-transfer (ToM) outcomes, the study seeks to contribute to a developmentally informed, ecologically grounded intervention model for children with SLD.

## 2. Materials and Methods

### 2.1. Participants

We recruited 40 pupils with a formal diagnosis of SLD (25 males and 15 females), aged between 8 and 10 years (Grades 3 & 4). Children were drawn from a larger study and were identified by three Greek mainstream primary schools, based on the interest expressed by specific schools that had inclusion classrooms for students presenting SLD. Participants were allocated to either the intervention group (*n* = 24) or a passive control group (*n* = 16). Because the intervention was implemented within the classroom setting, allocation occurred at the school rather than the individual participant level, to prevent cross-contamination between groups. As a result, the study employed a non-randomized, cluster-based quasi-experimental design.

All children held an official state diagnosis of SLD and were receiving Individualized Educational Programs (IEPs) within their schools. Official diagnostic reports and standardized learning assessment results were collected to verify the accuracy of each child’s diagnosis and to document their SLD profile. Children presented SLD profiles consistent with reading, written expression, and/or mathematics difficulties, as documented in their official diagnostic reports. Due to sample size constraints and the exploratory nature of the study, SLD subtypes were not analyzed separately; the sample was treated as a clinically homogeneous group, following previous intervention studies in SLD populations [22]. In addition, previous literature shows no significant differences emerging in the level of EF impairment [33].

To corroborate the official diagnoses, the Learning Disabilities Diagnostic Software (LAMDA) [34] was also administered to all participating children. LAMDA test is a software for the detection of specific learning difficulties within the general student population. Pupils’ performance is classified into 4 zones, compared to their classmates’ performance. To be included in the study, pupils had to perform significantly lower that their classmates in two or more areas of skills.

Children in both groups met the additional inclusion criteria: Greek was their first language, and they achieved an intelligence score within the normal range (IQ > 80), as measured on a standardized measure of non-verbal reasoning, the Raven’s Colored Progressive Matrices, Greek version [35,36]. Table 1 presents the demographic characteristics of the sample. The two groups did not differ significantly in gender, age, grade, or IQ.

### 2.2. Outcome Measures

Assessments were conducted at two time points: prior to the start of the intervention (T0) and immediately after its completion (T1). All evaluations were carried out individually by a licensed psychologist in a quiet room within the school premises. The assessment battery included performance-based measures of cool and hot EF and ToM and was administered in a fixed order to ensure consistency across participants. The fixed task sequence was selected based on prior recommendations for minimizing variability in individual differences [37,38] and to maintain uniform session duration and task balance across domains.

#### 2.2.1. Cool Executive Functions

Cognitive flexibility was assessed using Berg’s Card Sorting Task from the Psychology Experiment Building Language (PEBL) platform [38]. This computerized adaptation of the Wisconsin Card Sorting Test (WCST) [39] requires participants to sort cards displaying different shapes, colors, and quantities into one of four piles according to a rule that changes after every 10 trials. Feedback is provided after each response, indicating whether the selection was correct or incorrect, but without revealing the sorting principle. The task measures the ability to adapt to shifting rules and to modify responses accordingly. The number of correct responses was recorded, and previous research has demonstrated high reliability for computerized versions of this task [40].

Planning ability was evaluated using the Tower of London (ToL) [41]. The task consists of two identical wooden boards placed side by side, each containing three vertical pegs and three colored balls (green, red, and blue). Participants are asked to reproduce specific target configurations of the balls in a limited number of moves, adhering to two rules: (a) each problem must be solved within the prescribed number of moves, and (b) only one ball may be moved at a time. A total of 12 planning problems were presented. Each correctly solved problem, completed according to the rules, earned one point.

Verbal working memory was assessed using the Forward and Backward Digit Span subtests of the Wechsler Intelligence Scale for Children—Fifth Edition [42]. In the forward condition, participants were instructed to repeat sequences of numbers in the exact order presented by the examiner, with sequence length increasing progressively. In the backward condition, participants repeated the numbers in reverse order. Each correct trial was awarded one point, and testing continued until the participant failed both trials in a given sequence length. Separate scores were calculated for the forward and backward conditions, and their sum represented the composite working memory score.

Inhibition was measured using the Go/No-Go task, specifically the “R and P” version available on the PEBL platform [38]. During the task, letters “P” and “R” appeared sequentially at the center of a black screen for 1500 ms each. In the first block, participants were instructed to press a designated key when the letter “P” appeared (Go trials) and to withhold responses when the letter “R” appeared (No-Go trials). In the second block, the instruction was reversed. Each block began with 10 practice trials, followed by 320 experimental trials. No feedback was given during testing. The number of commission errors responses on No-Go trials was recorded.

#### 2.2.2. Hot Executive Functions

Affective decision-making was assessed using a computerized version of the Iowa Gambling Task (IGT) [43], adapted from the Psychology Experiment Building Language (PEBL) platform [38]. In this task, participants make a series of 100 card selections from four decks labeled A, B, C, and D. Decks A and B are disadvantageous, yielding higher immediate gains but larger long-term losses, whereas decks C and D are advantageous, offering smaller short-term rewards but better long-term outcomes. In the standard configuration, decks A and B are associated with total net losses, and decks C and D with net gains. Specifically, within each set of 20 trials, decks A and B can yield potential wins of €1000, interspersed with losses of up to €1250; deck B produces less frequent but larger losses than deck A. Conversely, decks C and D yield potential gains of €500, with possible losses of up to €250; deck D involves fewer but greater losses compared to deck C. Performance was evaluated by calculating the net IGT score, obtained by subtracting the number of disadvantageous choices (A + B) from the number of advantageous choices (C + D) divided by the total number of 100 trials, following the procedure described in Verdejo-García et al. [44].

A modified version of the Delay of Gratification task [45] was used to measure capacity for delayed gratification. This adaptation of the classic “marshmallow test” was designed for school-aged children and included two modifications: (a) extending the maximum waiting time to 20 min and (b) embedding the task within a food preference context. Each child was tested individually in a quiet, distraction-free room. They first completed a brief questionnaire about food preferences (e.g., choosing between different breakfast cereals or soft drink sizes). Subsequently, they selected their preferred snack and portion size from several available options. The researcher then left the room, instructing the child to wait until her return to receive the larger portion or to ring a bell to end the waiting period and receive a smaller one instead. The primary measure was waiting time in seconds, with a maximum score of 1200 s (20 min). Longer waiting times reflected greater capacity for delayed gratification and self-control.

#### 2.2.3. Theory of Mind

Participants’ understanding of false beliefs was evaluated using the Sandbox Task [46]. In this task, children listened to a short story about a father and daughter planting flower bulbs in a sandbox while viewing an accompanying illustration. The father initially buried the bulb at a location marked with a cross before leaving the scene, after which the daughter secretly moved it to a new location. Participants were then asked to indicate, by drawing a cross, where the father would water the flower bulb upon his return (false-belief question). The distance (in centimeters) between the original hiding location (0 cm) and the location marked by the participant was recorded. Responses closer to the daughter’s new location (6.3 cm) were assigned positive bias scores, while responses in the opposite direction received negative scores. Lower scores reflected better performance. Only the false-belief condition of the Sandbox Task was used in this study. This task was selected to minimize ceiling effects commonly observed in traditional false-belief paradigms and to capture subtle individual differences in false-belief reasoning among older children. 

The Reading the Mind in the Eyes Test [47] was administered to assess participants’ ability to infer others’ mental and emotional states. The task consists of 28 photographs showing only the eye region of various individuals, each accompanied by four descriptive words. Participants were instructed to carefully examine each image and select the word that best describes what the person in the picture is thinking or feeling. Each correct answer was awarded one point, yielding a total possible score ranging from 0 to 28. The task demonstrated satisfactory test–retest reliability [48,49].

### 2.3. The Intervention

The intervention implemented in this study was based on the Unstuck and On Target! (UOT) curriculum [50,51]. UOT was delivered exclusively to the intervention group in small groups within the inclusion classroom setting during school hours by an experienced teacher. The program comprised a fixed total of 18 sessions for all participants. Sessions were delivered over approximately 6–9 weeks, with two to three 45–50 min sessions conducted per week depending on school scheduling constraints. Importantly, all children in the intervention group received the same number of sessions and identical intervention content; variability reflected differences in scheduling density rather than differences in intervention dose. Children in the control group continued to participate in their regular school curriculum and daily classroom routines, which typically included group-based learning tasks, reading, writing, maths and problem-solving activities appropriate for their grade level. These activities did not explicitly target EF or ToM skills. An active control condition was not implemented due to constraints related to school scheduling, staffing availability, and the need to avoid additional instructional burden on participating classrooms.

Grounded in the principles of cognitive–behavioral therapy, UOT is designed to teach children aged 8–11 years critical skills related to EFs, such as cognitive flexibility, planning, goal setting, and self-regulation. The curriculum was originally designed for primary school children on the autism spectrum and shown to effectively strengthen EF [52]. Although the Unstuck and On Target (UOT) curriculum was originally developed and first evaluated with children on the autism spectrum [52], the updated version of the program is explicitly designed to support children with executive-function difficulties more broadly [51], across neurodevelopmental and educational profiles [53]. This broader conceptualization aligns well with the EF characteristics commonly observed in children with SLD, who often present with difficulties in planning, organization, cognitive flexibility, and academic self-regulation, rather than with core social-communication impairments. The curriculum manual of UOT [52] comprises 21 structured, group-based, ready-to-use lessons designed to promote EF in everyday contexts, such as negotiating with peers or coping with unexpected schedule changes. Program activities target the development of flexible thinking, goal-directed behavior, self-organization, and the use of internalized self-talk for problem solving. Lessons are delivered through interactive and experiential methods, including hands-on experiments (e.g., comparing performance on an obstacle course using a “rigid” versus “flexible” body), videos, visual aids, and guided discussions. The curriculum also includes self-regulatory scripts (e.g., Plan A/Plan B, Big Deal/Little Deal) which are practiced through creative activities (e.g., designing a pretend video game with flexibility challenges and solutions), structured games, and role-play exercises. Motivation to apply flexible and goal-oriented strategies is further supported through discussions and games that emphasize the advantages of flexibility, goal setting, and coping skills.

In the present study, the curriculum was implemented with an emphasis on executive processes particularly relevant to learning in classroom contexts, including goal management, flexible problem solving, task persistence, and the use of self-regulatory strategies to cope with academic demands. Activities were framed around learning-related challenges (e.g., completing multi-step tasks, adapting to changes in classroom routines, managing frustration during problem solving), ensuring developmental and cognitive appropriateness for children with SLD while preserving the theoretical foundations of the original curriculum. Although the curriculum primarily targets cool executive functions, its small-group, interactive format incorporates activities that introduce affective and social-cognitive demands. Group-based problem solving, role-play, peer discussion, and guided reflection require children to negotiate goals, regulate emotional responses, and consider others’ perspectives in socially meaningful situations. These elements were not intended to directly train hot executive functions or Theory of Mind; rather, they were designed to provide opportunities for indirect engagement of motivational, emotional, and socio-cognitive processes within authentic classroom interactions.

Eighteen (18) out of the 21 lessons from the UOT manual were selected for implementation to ensure feasibility within the Greek school context and to align the overall duration of the program with the available instructional time. The lessons that were omitted either presented overlapping instructional objectives or included culturally specific elements that were not considered fully appropriate for the Greek educational environment. All instructional materials and activities were translated and culturally adapted into Greek using standard translation and back-translation procedures to preserve conceptual equivalence with the original content.

Prior to implementation, the teacher who delivered the intervention received training in the curriculum. Training was conducted by a licensed psychologist and a special educator familiar with the program’s theoretical foundations and instructional methods. The teacher was introduced to the core principles of cognitive–behavioral instruction, the use of self-regulatory scripts, and the step-by-step delivery of each lesson. Throughout the intervention period, implementation fidelity was monitored through weekly supervision meetings and the completion of standardized fidelity checklists after each session to ensure consistency and adherence to the program protocol.

### 2.4. Data Analysis

All statistical analyses were performed using the IBM Statistical Package for the Social Sciences (SPSS), Version 26. Prior to conducting inferential analyses, the assumptions of normality and homogeneity were examined. For within-group comparisons (pre- and post-intervention), paired-samples *t*-tests were performed for variables that met the normality assumption, while the non-parametric Wilcoxon signed-rank test was applied to variables that did not. For between-group baseline comparisons, independent-samples t-tests and Mann–Whitney U tests were used as appropriate. To further assess the effectiveness of the intervention while controlling for initial group differences, analyses of covariance (ANCOVA) were performed using pre-test scores as covariates [54]. When ANCOVA assumptions were violated, the Quade non-parametric ANCOVA [55] was employed. Effect sizes (η^2^) were computed to evaluate the strength of the observed effects. Based on Cohen’s [56] conventions, values around 0.01 indicate small effects, 0.06 medium effects, and 0.14 large effects.

## 3. Results

### 3.1. Within-Group Differences (Before and After Intervention)

Within-group comparisons were conducted to examine changes in performance between the pre- and post-assessments for children with SLD in both the control and intervention groups. For the control group (see Table 2), no statistically significant differences were observed across measures of cool or hot EF or ToM (*p* > 0.05). Although slight improvements were noted in planning and cognitive flexibility scores, these differences did not reach statistical significance. Similarly, small, non-significant changes were found in affective decision-making, delay of gratification, and ToM tasks. Overall, the control group’s performance remained stable across the two assessment points, indicating no improvement in the absence of intervention.

For the intervention group (Table 3), the analyses revealed statistically significant improvements in working memory (*t*(23) = −2.145, *p* = 0.043), planning (*t*(23) = −2.571, *p* = 0.017), and cognitive flexibility (*t*(23) = −3.168, *p* = 0.004). These results suggest that participation in the intervention was associated with enhanced performance in key components of cool EF. Although a marginal improvement was observed in ToM mental state/emotion recognition (*t*(23) = −1.978, *p* = 0.060), this difference did not reach statistical significance. No significant changes were found in inhibition, affective decision-making, delay of gratification, or false belief understanding (all *p* > 0.05). Taken together, these findings indicate that participation in the intervention was associated with improvements in cool EF, with limited evidence of change in hot EF and ToM abilities.

### 3.2. Baseline (Pre-Intervention) Group Comparisons

Baseline comparisons between the control and intervention groups were conducted using independent-samples *t*-tests and Mann–Whitney *U* tests, depending on data distribution (Table 4). Overall, the analyses indicated no significant between-group differences across most measures of cool EF, hot EF, or ToM (*p* > 0.05), suggesting that the groups were largely comparable prior to the intervention. However, two significant baseline differences emerged. The intervention group performed better on false belief understanding (*Z* = −2.988, *p* = 0.003), whereas the control group showed higher accuracy in inhibition (*Z* = −2.748, *p* = 0.006). These differences are taken into account when interpreting subsequent post-intervention effects.

### 3.3. Post-Intervention Comparisons (Efficacy of the Training)

**Figure 1:**
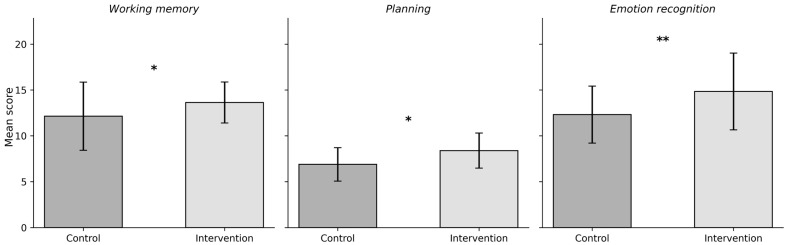
Group differences in working memory, planning, and ToM mental state/emotion recognition at post-assessment. Error bars represent standard deviations (SD). Note. * p < 0.05, ** *p* < 0.01.

To account for baseline differences and to evaluate the effectiveness of the intervention, analyses of covariance (ANCOVA) were conducted with pre-test scores entered as covariates (Table 5). This approach, which integrates regression and analysis of variance, is recommended for experimental designs using pre–post assessment structures [55]. The results indicated significant group effects for working memory (*F* (1, 38) = 5.620, *p* = 0.023, η^2^ = 0.129), planning (*F* (1, 38) = 4.626, *p* = 0.038, η^2^ = 0.109), and ToM mental state/emotion recognition (*F* (1, 38) = 9.716, *p*= 0.003, η^2^ = 0.204) (Figure 1). Based on Cohen’s [57] guidelines, these represent medium-to-large effect sizes, suggesting that children who participated in the intervention showed meaningful improvements in these domains relative to the control group. No significant group differences were found for inhibition, cognitive flexibility, affective decision-making, delay of gratification, or false belief understanding (*p* > 0.05).

## 4. Discussion

This study examined the effectiveness of a structured, classroom-based EF program in enhancing cool and hot EF skills and ToM in primary-school children with SLD. Consistent with our hypotheses, the intervention was associated with significant improvements in several cool EF domains, specifically working memory and planning, alongside within-group gains in cognitive flexibility. In contrast, no significant between-group effects were found for hot EF components (affective decision-making and delay of gratification) or for cool inhibition, indicating that these domains may require more intensive, explicit, or longer-lasting training to improve. Importantly, the intervention showed a significant between-group improvement in ToM mental state/emotion recognition, providing evidence of far transfer to socio-cognitive processes. Together, these findings suggest selective improvement in specific core cool EF skills and enhanced aspects of ToM understanding, while having no significant short-term impact on hot EF domains. These effects emerged despite the task–training heterogeneity inherent to performance-based measures, lending cautious support to the inference that improvements reflect genuine cognitive and socio-cognitive change rather than task-specific practice. Given the non-randomized, cluster-based design of the study, these findings should be interpreted as associations consistent with participation in the intervention rather than as evidence of direct causality.

The intervention group exhibited greater post-intervention improvement than the control group in three cool EF domains. Importantly, improvements were not generalized across all EF domains, suggesting that the intervention selectively engaged EF processes most directly targeted by the curriculum, rather than producing broad executive enhancement. Gains in working memory were particularly notable and align with a growing body of research demonstrating that EF training enhances working-memory capacity and attentional control in children with learning difficulties. For example, Chavez-Arana et al. [24] reported significant improvements in both verbal and visuospatial working memory following EF-focused training, while Takacs and Kassai’s meta-analysis [30] highlighted working memory as one of the most responsive EF components to structured, self-regulation-based interventions in neurodivergent populations. Similar benefits have been observed in children with autism spectrum disorder following the UOT program [52], and results from subsequent studies [57,58] further corroborate that EF-focused curricula can meaningfully strengthen working-memory updating and monitoring processes. Collectively, these findings suggest that working memory is highly malleable in middle childhood, particularly when strengthened through collaborative, goal-oriented activities that require continuous updating and reflection.

Planning also improved significantly more in the intervention group than in the control group. This aligns with previous evidence showing that direct training in planning strategies enhances problem-solving skills and organizational behaviors across neurodevelopmental conditions [30,53]. Improved performance on the Tower of London task suggests that the intervention strengthened children’s ability to anticipate steps, sequence actions, and monitor progress, skills that underpin academic tasks such as writing, mathematical problem solving, and coordinating multi-step classroom activities. The program’s structured, group-based format may have further supported planning by encouraging children to articulate their strategies, negotiate roles, and reflect on their performance.

Cognitive flexibility showed small-to-moderate improvement despite nonsignificant between-group differences, providing partial support for the curriculum’s efficacy. Flexibility is known to develop more gradually than other cool EF components and follows a protracted developmental trajectory extending into late childhood and adolescence [7,59,60,61]. It is also highly context dependent and particularly sensitive to task demands and environmental structure, making it less responsive to short-term interventions compared with working memory or planning [4,5]. Measurement characteristics may also have constrained detection of transfer effects. Tools such as the BCST assess abstract rule shifting in controlled contexts but may not reflect the form of flexible thinking typically required in peer negotiation or spontaneous classroom adjustment. Thus, flexibility may have begun to develop but was not yet detectable via standard performance-based metrics. Nevertheless, even modest improvements are meaningful in classroom settings, where flexible behavior, such as shifting strategies, adapting to new rules, and navigating unpredictable social interactions, plays a critical role in successful learning and social adjustment. Future studies using ecologically valid measures, with longer training duration or dynamic flexibility tasks may be better positioned to detect emerging changes in this domain.

No significant between-group effects were found for hot EF (affective decision-making and delay of gratification) or for cool inhibition. Several explanations are plausible. First, although the intervention incorporated elements related to behavioral regulation, these represented a limited proportion of the overall training dosage, potentially limiting training dosage for these processes. Research suggests that inhibition and reward-based regulation require frequent, explicit, and sustained practice to produce meaningful change, particularly among children with neurodevelopmental conditions [3]. Moreover, inhibitory skills follow a relatively protracted developmental trajectory linked to the maturation of prefrontal–striatal circuitry [62,63,64], making them less sensitive to brief, classroom-based interventions. Measurement limitations may also account for the null findings. Laboratory-based hot EF tasks capture specific components of affective and reward decision-making under controlled conditions; however, they may not fully represent how children regulate emotions or motivation in socially embedded, classroom contexts. Since hot EF is strongly modulated by affective and motivational salience [64], future research may benefit from complementing standardized tasks with ecologically valid, socially interactive assessments to capture a broader range of regulatory processes.

Even though findings related to Theory of Mind should be interpreted cautiously and as exploratory, given that ToM was not directly trained and effects were limited to mental state/emotion recognition, a distinctive contribution of this study was the examination of far transfer to ToM abilities. The intervention group demonstrated greater post-intervention improvement in mental state/emotion recognition, extending recent findings linking EF and ToM in children with SLD [11] and supporting theoretical accounts positing that cool EF components such as working memory and inhibition scaffold children’s ability to represent, coordinate, and reason about mental states [13,65]. Improvements in mental state/emotion recognition also align with evidence from meta-analytic work showing that EF-focused, self-regulation-based interventions can support emotional understanding and social-cognitive flexibility in neurodivergent populations [30]. Mechanistically, the program’s emphasis on goal setting, perspective shifting, metacognitive monitoring, and collaborative problem solving likely activated cognitive processes central to mental-state reasoning. The interactive group activities required children to coordinate actions, anticipate peers’ responses, and interpret emotional signals, thereby providing repeated, meaningful opportunities to practice socio-cognitive skills within authentic social contexts. Such dynamics are consistent with findings that EF-based interventions can be associated with improvements in ToM even when social cognition is not explicitly taught [66,67], highlighting the potential for indirect, yet meaningful, socio-cognitive gains through executive-focused instruction.

While significant gains were found in mental state/emotion recognition (Reading the Mind in the Eyes), no measurable improvement was observed in false-belief reasoning (Sandbox Task). This discrepancy may reflect differences in task complexity and developmental sensitivity. More specifically, the Sandbox Task requires children to integrate visuospatial memory, belief reasoning, and inhibitory control simultaneously, perhaps making it less sensitive to short-term intervention effects. Moreover, middle childhood is a period in which standard false-belief tasks may often approach a developmental ceiling, especially in verbally able children, reducing between-subject variability and limiting the possibility of observable change over brief intervention periods. Although the use of the Sandbox Task was driven by the need to capture subtle individual differences beyond traditional paradigms, results suggest that yet even this task may present reduced sensitivity to short-term interventions in older school-aged samples.

The findings contribute to theoretical models and studies that conceptualize cool executive functions as an important, but not sufficient, component supporting socio-cognitive development [68,69]. Rather than implying a direct or uniform causal pathway, contemporary developmental frameworks emphasize that EF and social cognition are partially overlapping, dynamically interacting systems whose relations vary across developmental stages and contexts. Within this perspective, EF capacities such as working memory, inhibition, and cognitive flexibility may support children’s engagement in social-cognitive reasoning by enabling them to hold, coordinate, and reflect on mental-state representations [65], without constituting a deterministic developmental sequence. These models propose that executive control processes may facilitate, among other things, the suppression of one’s own perspective and the consideration of alternative viewpoints; processes that are central to mental-state reasoning but that operate in conjunction with linguistic, emotional, and contextual factors. The present findings are consistent with this view, suggesting that participation in EF-focused, collaborative learning activities may create conditions that support certain aspects of socio-cognitive processing, rather than generalized or robust transfer to all ToM components. The results further highlight the interrelated nature of EF and social cognition in ecologically valid contexts; structured, collaborative classroom activities impose simultaneous demands on cognitive control, joint attention, emotional regulation, and interpersonal understanding, thereby engaging overlapping neural and cognitive systems. Collectively, these insights align with domain-general and neuroconstructivist accounts of EF development, which propose that executive processes can have cascading, context-dependent influences on social understanding, emotional reasoning, and adaptive behavior in everyday interactions, particularly when embedded in meaningful social environments [69,70].

An important conceptual and applied contribution of the present study lies in examining the application of the UOT curriculum in children with SLD, a population for whom structured, classroom-based executive-function interventions remain relatively underexplored. Although the curriculum is explicitly designed to support children with executive-function challenges across developmental and educational profiles [51], empirical evidence regarding its use in SLD populations is limited. The present findings suggest that an EF curriculum grounded in flexible thinking, planning, and self-regulation may be suitable for use with children with SLD when embedded within inclusive classroom environments and aligned with learning-related executive demands. This contribution supports the broader applicability of EF-focused interventions across neurodevelopmental profiles, while underscoring the importance of population-sensitive implementation.

The findings have several implications for educational practice from a neurocognitive perspective. Integrating EF instruction into everyday classroom routines provides children with repeated opportunities to activate and strengthen fronto-parietal and fronto-striatal networks that support planning, working memory, and cognitive flexibility. When EF strategies are practiced within every day, socially interactive learning activities, such as collaborative problem solving, goal-setting discussions, and perspective-taking tasks, children are more likely to generalize these skills, as they are engaged in contexts that mirror naturalistic demands on executive control [3,4]. Embedding EF supports within regular instruction is also consistent with neuro-constructivist and socio-constructivist models of development, which emphasize that cognitive growth is shaped by structured, scaffolded experiences embedded in meaningful environments [70]. From an applied standpoint, classroom-based EF interventions are feasible, scalable, and compatible with inclusive education frameworks, allowing educators to implement developmentally informed supports without removing children from their primary learning environment. This is particularly relevant for children with SLD, whose difficulties in strategic behavior, organization, and self-regulation often reflect underlying EF vulnerabilities that impede academic and social functioning. By targeting core executive processes that underlie engagement, task persistence, and social interaction, EF-focused classroom programs may enhance both learning outcomes and adaptive behavior across the school day.

Several limitations should be acknowledged. First, the sample included only children in Grades 3–4, which restricts the generalizability of the findings to other developmental stages. Second, the quasi-experimental, cluster-based design did not allow for individual-level randomization. Although allocation at the school level minimized contamination, it may have introduced unmeasured school-level effects. As a result, the distribution of SLD subtypes which may have differed between groups could have influenced performance profiles and should be addressed in future research through stratified or cluster-randomized designs. Third, the modest sample size limited statistical power, particularly for detecting smaller effects in hot EF domains, and may also have affected the precision and generalizability of the observed effects. Effect size estimates derived from small samples are known to be less stable and more sensitive to sampling variability and therefore should be interpreted with appropriate caution. While the large effects observed are encouraging, replication in larger, adequately powered and more diverse samples is necessary to establish the robustness of these findings and to better capture potentially smaller effects in complex domains such as hot executive function and false-belief reasoning. Fourth, outcome measures relied primarily on performance-based tasks, which may not fully capture children’s everyday executive or socio-emotional functioning. Incorporating teacher- and parent-report measures, classroom observations, and ecologically valid behavioral indices would provide a more comprehensive assessment. An additional limitation concerns the nature of the control condition. The control group followed the usual school curriculum, whereas children in the intervention group received additional structured, small-group sessions. As a result, it is not possible to disentangle the specific effects of EF content from non-specific factors such as increased adult attention, expectancy effects, group structure, or engagement associated with participation in a novel, structured program. Consequently, the observed group differences should be interpreted as reflecting effects associated with participation in the intervention, rather than as evidence of content-specific EF mechanisms. Future studies should address this limitation by incorporating active control conditions to more precisely isolate the mechanisms underlying EF-focused interventions. Moreover, although all participants received the same total number of intervention sessions, variability in the temporal distribution of sessions across weeks may have influenced individual responsiveness to the intervention and should be considered when interpreting the findings. In addition, although intervention fidelity was supported through teacher training, supervision, and session checklists, quantitative fidelity indices (e.g., adherence rates) were not calculated, which limits more fine-grained evaluation of implementation consistency. Finally, the absence of follow-up data prevents conclusions about the durability of training effects. Longitudinal assessments are needed to determine whether gains in EF and ToM translate into sustained improvements in academic and social outcomes. These methodological features constrain the strength of inferences that can be drawn regarding specificity and causality and may partly account for the selective pattern of findings observed.

## 5. Conclusions

Overall, children in the intervention group showed meaningful gains in several cool EF domains and in one component of ToM, specifically mental state/emotion recognition, relative to the control group. Taken together, these findings are consistent with the view that participation in school-embedded EF instruction may be associated with improvements in both cognitive and selected socio-cognitive processes in children with SLD, supporting the broader notion that executive processes remain malleable during middle childhood. Importantly, the integration of the program into everyday classroom routines highlights the potential feasibility of supporting EF development within naturalistic educational settings, without reliance on specialized clinical environments. Rather than demonstrating direct causal effects, the present findings suggest that developmentally attuned, collaborative, and contextually grounded classroom activities may provide conditions under which executive and socio-cognitive skills can be engaged and practiced. The observed differences in mental state/emotion recognition should be interpreted cautiously and as exploratory, given the absence of effects on other Theory of Mind components. Nonetheless, these results align with developmental models emphasizing the interconnectedness of executive control and social cognition and point toward a promising direction for future research examining how school-based, ecologically valid interventions may support learning and adjustment in children with SLD.

## Figures and Tables

**Table 1:** Demographic Characteristics of the Intervention and Control Groups.

Child Characteristic		Control (*n* = 16) *M* (SD)	Intervention (*n* = 24) *M* (SD)	Statistic	*p*
Gender	Boy	62.5% (10)	62.5% (15)	χ^2^(1) = 0.000	0.99
	Girl	37.5% (6)	37.5% (9)		
Age	–	9.28 (0.73)	8.86 (0.62)	*t*(38) = 1.947	0.059
Grade	3rd	50.0% (8)	58.3% (14)	χ^2^(1) = 0.269	0.604
	4th	50.0% (8)	41.7% (10)		
IQ	–	94.69 (11.76)	100.21 (13.23)	*Z* = −1.329	0.184

Note. χ^2^ = chi-square test of independence; *Z* = Mann–Whitney test; *t* = independent samples *t*-test.

**Table 2:** Comparison of pre- and post-intervention performance in the control group.

	Pre *M* (SD)	Post *M* (SD)	Statistic	*p*
Cool Executive Functions				
Inhibition errors	238.06 (14.60)	243.13 (15.57)	*Z* = −1.009	0.313
Working memory	13.13 (3.54)	12.13 (3.72)	*t*(15) = 1.426	0.174
Planning	6.25 (1.44)	6.88 (1.82)	*t*(15) = −1.373	0.190
Cognitive flexibility	67.75 (20.15)	70.19 (21.43)	*t*(15) = −0.689	0.469
Hot Executive Functions				
Affective decision-making	−0.050 (0.13)	−0.023 (0.19)	*t*(15) = −0.734	0.459
Delay of gratification	915.25 (415.62)	960.13 (347.00)	*Z* = −0.357	0.721
Theory of Mind (ToM)				
False belief understanding	2.98 (2.73)	1.96 (3.15)	*Z* = −1.643	0.100
Mental state/emotion recognition	14.00 (4.16)	12.31 (3.11)	*t*(15) = 2.097	0.053

Note. *Z* = Wilcoxon signed-rank test; *t* = paired-samples *t*-test.

**Table 3:** Comparison of Pre- and Post-Intervention Performance in the Intervention Group.

Variable	Pre *M* (SD)	Post *M* (SD)	Statistic	*p*
Cool Executive Functions				
Inhibition errors	253.92 (23.20)	256.58 (17.98)	*Z* = −0.686	0.493
Working memory	12.79 (2.78)	13.63 (2.24)	*t*(23) = −2.145	0.043 *
Planning	7.13 (1.70)	8.38 (1.91)	*t*(23) = −2.571	0.017 *
Cognitive flexibility	79.21 (12.69)	85.79 (13.13)	*t*(23) = −3.168	0.004 **
Hot Executive Functions				
Affective decision-making	–0.060 (0.30)	–0.086 (0.22)	*Z* = −0.167	0.867
Delay of gratification	939.79 (344.10)	951.63 (369.51)	*Z* = −0.454	0.650
Theory of Mind (ToM)				
False belief understanding	1.04 (2.61)	1.47 (2.58)	*Z* = −0.601	0.548
Mental state/emotion recognition	13.58 (4.83)	14.83 (4.19)	*t*(23) = −1.978	0.060

Note. *Z* = Wilcoxon signed-rank test; *t* = paired-samples *t*-test; * *p* < 0.05; ** *p* < 0.01.

**Table 4:** Baseline comparisons between the control and intervention groups.

Variable	Control *M* (SD)	Intervention *M* (SD)	Statistic	*p*
Cool Executive Functions				
Inhibition errors	238.06 (14.60)	253.92 (23.20)	*Z* = −2.748	0.006 **
Working memory	13.13 (3.54)	12.79 (2.78)	*t*(38) = 0.333	0.741
Planning	6.25 (1.44)	7.13 (1.70)	*t*(38) = −1.691	0.099
Cognitive flexibility	67.75 (20.15)	79.21 (12.69)	*t*(22.93) = −2.022	0.055
Hot Executive Functions				
Affective decision-making	−0.05 (0.13)	−0.06 (0.30)	*Z* = −0.277	0.782
Delay of gratification	915.25 (415.62)	939.79 (344.10)	*Z* = −0.090	0.929
Theory of Mind (ToM)				
False belief understanding	2.98 (2.73)	1.04 (2.61)	*Z* = −2.988	0.003 **
Mental state/emotion recognition	14.00 (4.16)	13.58 (4.83)	*t*(38) = 0.282	0.779

Note. *Z* = Mann–Whitney test; *t* = independent-samples *t*-test; ** *p* < 0.01.

**Table 5:** Comparison of the post-intervention performances of the control and intervention groups on executive function and Theory of Mind tasks.

Variable	Control *M* (SD)	Intervention *M* (SD)	*F* (1, 38)	*p*	η^2^
Cool Executive Functions					
Inhibition errors	243.13 (15.57)	256.58 (17.98)	1.924	0.173	0.048
Working memory	12.13 (3.72)	13.63 (2.24)	5.620	0.023 *	0.129
Planning	6.88 (1.82)	8.38 (1.91)	4.626	0.038 *	0.109
Cognitive flexibility	70.19 (21.43)	85.79 (13.13)	2.368	0.132	0.059
Hot Executive Functions					
Affective decision-making	−0.023 (0.19)	−0.086 (0.22)	1.034	0.316	0.026
Delay of gratification	960.13 (347.00)	951.63 (369.51)	0.007	0.933	<0.001
Theory of Mind (ToM)					
False belief understanding	1.96 (3.15)	1.47 (2.58)	1.062	0.309	0.027
Mental state/emotion recognition	12.31 (3.11)	14.83 (4.19)	9.716	0.003 **	0.204

Note: * *p* < 0.05; ** *p* < 0.01.

## Data Availability

The datasets generated and analyzed during the current study is available from the corresponding author upon reasonable request due to privacy.

## Abbreviations

The following abbreviations are used in this manuscript:

EF	Executive function
ToM	Theory of Mind
SLD	Specific Learning Disorders
UOT	Unstuck and On Target
